# The Histological Components of the Phoniatrical Body-Cover Model in Minipigs of Different Ages

**DOI:** 10.1371/journal.pone.0128085

**Published:** 2015-05-27

**Authors:** Anja Lang, Rüdiger Koch, Karl Rohn, Hagen Gasse

**Affiliations:** 1 Institute of Anatomy, University of Veterinary Medicine Hannover, Hanover, Germany; 2 Department of Biometry, Epidemiology and Information Processing, University of Veterinary Medicine Hannover, Hanover, Germany; Research Center Borstel, GERMANY

## Abstract

Pigs are models in human phoniatry. However, features of maturation and ageing have not been considered with regard to the so-called body-cover model in this species. Therefore, the glottis of “young” (2–3 months; n = 6) and “old” (4–7 years; n = 6) minipigs was investigated. Their cranial (CraF) and caudal (CauF) vocal folds were histomorphometrically and stratigraphically analysed with emphasis on their amounts of collagen structures and elastic fibres. A dense subepithelial layer (SEL) was a distinct feature of CraF and CauF of both age groups; it was spread upon the underlying loose, flexible “cover” like a fibro-elastic membrane. The “cover” was characterised by the so-called superficial layer (SL), which was distinctly loose in the “young” minipigs, but had a much denser texture in the “old” minipigs. Here, the SL was dominated by elastic fibres in the CraF, but was of mixed qualities (collagenous and elastic) in the CauF. The structural requirements for the SL’s function as a loose “cover” were thus met only in the “young” animals. A clearly demarcated intermediate layer (IL)—characterised by high amounts of elastic fibres (as in humans)—was only found in the CraF of the “young” animals. In the “old” animals, it had lost its demarcation. In the depth of the CraF of the “old” animals, many thick collagen fibre bundles were detected in a location equivalent to that of the vocal muscle in the CauF. The development of their large diameters was interpreted as part of the maturation process, thereby supporting the hypothesis of their functional importance as a component of the “body.” In the CauF, the amounts of collagen structures increased throughout the entire lamina propria, resulting in a loss of demarcated stratigraphical subdivisions in the “old” minipigs. This situation resembled that described in the vocal fold of geriatric humans.

## Introduction

The mature human vocal fold possesses a specific stratigraphical organisation, which is chiefly characterised by connective tissue fibres [1]. The specific types, contents, and arrangements of these fibres, along with the Ground Substance, determine the biomechanical properties of the tissue [2–3]. These properties are implemented in the so-called body-cover model of vocal fold vibration [4]. According to this model, there is a ‘cover’ consisting of the epithelium and the superficial part of the vocal fold’s lamina propria. This ‘cover’ is soft and pliant as Ground Substance is its main component; fibres are relatively sparse. The ‘cover’ moves as a surface wave upon the fairly rigid ‘body’ [4–5]. The ‘body’ is made up of two components: Firstly, of the deeper part of the lamina propria containing large amounts of elastic fibres and collagen fibres, and secondly, of the vocal muscle. There is no unanimous agreement amongst authors as to the precise assignment of histological layers to either ‘body’ or ‘cover’, and it appears controversial in which layer the ‘cover’ ends and the ‘body’ begins (see e.g. [1–2, 4, 6–7]).

Pigs are established models in human phoniatry [8–14]. Nonetheless, care has to be taken to respect the specific anatomy of the porcine glottis [15–16]. There are two very different vocal folds on either side of the larynx, i.e. a caudal fold (CauF) and a cranial fold (CraF). The CauF seems to resemble the human vocal fold in terms of topography, and the above-mentioned human body-cover model has also been proposed for the CauF of the pig [8]. Yet, the CauF is not the main oscillator; Alipour and Jaiswal [12–13] assigned this function to the CraF.

The CraF, despite its different localisation, and despite its lack of a proper vocal muscle, appears histologically very similar to the human vocal fold [15]. This is true, in particular, as far as elastic fibres are concerned [15]. Considering the histological/stratigraphical elements of a body-cover model, Lang et al. [15] have suggested that this model may also apply for the CraF. Consequently, the lack of a vocal muscle—a part of the ‘body’ of the CauF—may be substituted by the large amounts of thick collagen fibre bundles present in the respective proportional depth of the CraF.

During maturation and ageing, the lamina propria of the human vocal fold is subject to extensive changes affecting its biomechanics and oscillatory behaviour [2]. In newborns, the lamina propria shows a homogeneous composition of few fibres and abundant Ground Substance [17–18]. At this age, the entire lamina propria acts as a soft, loose ‘cover’ during oscillation, while the vocal muscle alone forms the ‘body’ [19].

Ageing (senescence) of the vocal fold’s lamina propria affects the microstructure of fibres, as well as their amounts and distribution [7, 20–24]. These alterations also have an impact on the biomechanical properties of ‘cover’ and ‘body’, and on their dimensions; e.g. in humans, there is an increase in the thickness of the ‘cover’ with age [19], contributing to the ageing of the voice [20, 25].

Despite a considerable amount of information on the stratigraphical composition of the vocal folds of pigs [8–9, 15, 26–28], there are no data available to demonstrate if relevant structural changes occur with maturation and ageing also in the glottis of pigs. Blakeslee et al. [8] described the stratigraphy of the CauF as resembling that of a child. However, no precise information was given on the age of the examined animals—a problem frequently encountered in the literature. Moreover, the life-span and the speed of maturation and ageing differ significantly between pigs and humans [29–30]. With regard to the great impact of the histological structure on the phoniatrical function in humans, any attempt to evaluate the pig’s true potential as an animal model will remain insufficient as long as age-related changes have not been evaluated. The present study attempts to provide such data by using a recently established histomorphometrical procedure [15–16].

## Materials and Methods

### (1) Ethics Statement and Specimens

Minipigs of the age groups ‘young’ (2–3 months; n = 6) and ‘old’ (4–7 years; n = 6) were included in this study. The status ‘young’ was assigned to the 2–3 month-old animals as they had not yet reached sexual maturity, which occurs at 4–6 months in minipigs [31]. The 54–85 month-old (4–7 year-old) animals were classified as ‘old’ because, firstly, minipigs reach adulthood in terms of weight development at approx. 2 years [29], secondly, epiphyseal closure in the long bones in completed at approximately 1.5–3 years [30], and finally, their life span is approx. 10–15 years [29]. All minipigs were female, and their breed was either Göttinger Minipig, Göttinger Minipig x Minnesota Minipig, or Mini Lewe Minipig. Minipigs were chosen for this study as they have become a common type of pig in animal experimentation [31–32] due to their closer similarity to humans in size and easier handling compared to much larger domestic pigs.

The minipigs were obtained from local farmers or animal trade companies. When the animals arrived at the institute’s facilities, they were not housed, but euthanised immediately after arrival. The German Federal Law, i.e. Protection of Animals Act (Tierschutzgesetz § 4, § 7, § 7a) and the Guidelines of the European Convention for the Protection of Vertebrate Animals used for Experimental and other Scientific Purposes (86/609/EEC) were observed. Accordingly, notice of the minipigs’ euthanasia for the purpose of organ removal from the dead animals (for subsequent anatomical and histological studies) was given to The Animal Welfare Officer of the University of Veterinary Medicine Hannover ahead of the study. In a second step, this report was reconfirmed in the annual reports on all animals used within the period of the respective year. According to the German Federal Law, i.e. Protection of Animals Act (Tierschutzgesetz § 7), an explicit permission to perform this study was not necessary because no medical procedures or experiments were performed while the animals were alive, except intravenous injection for euthanasia. Euthanasia was performed by qualified and authorised staff by means of intravenous injection of 1.5 ml/10 kg Euthadorm (Pentobarbital Sodium; CP-Pharma Handelsgesellschaft mbH, Burgdorf, Germany) or by captive bolt stunning and consecutive bleeding.

### (2) Histological procedures

**Fig 1:**
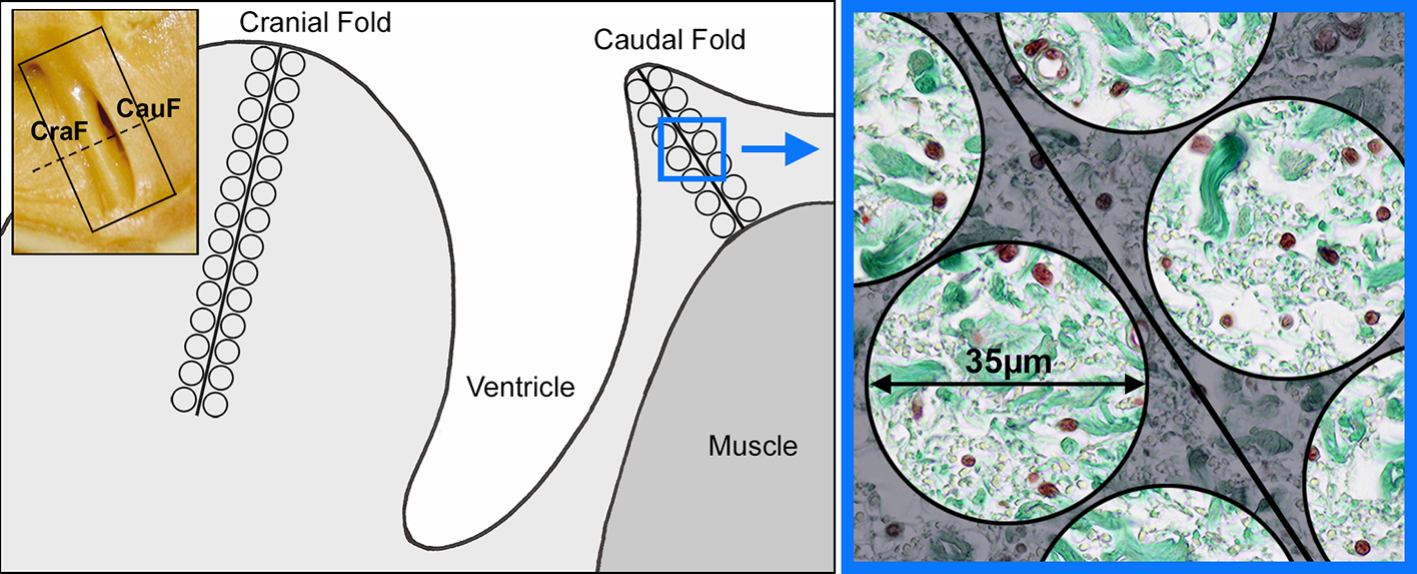
Graphical illustration of a cross section (coronal section) of the porcine glottis demonstrating the cranial and caudal fold, CraF and CauF, and pairs of ROIs placed along the midlines of the CraF and CauF (from Lang [16], modified). The midline through the CraF was twice as long as the midline through the neighbouring CauF; the diameter of each ROI was 35 μm. For the scoring procedure, however, the diameter of the ROIs was 70 μm. Inset upper left: Cranial and caudal fold of the fixed glottis; the dashed line indicates where the midportion was transversally cut (coronal sections) prior to placing the two resulting tissue blocks in the embedding moulds.

The larynges of the minipigs were immersion-fixed in Bouin’s solution. After fixation, one side of the glottis was transversally cut in its midportion, as illustrated in Fig 1. Then, the cut surface of the two resulting tissue blocks was placed at the bottom of the paraffin embedding moulds. This procedure ensured that the tissue blocks were always placed in identical positions, facilitating the processing of standardised coronal sections of the midportion of the folds, which is a frequently used procedure [7–8, 11, 20, 22, 24]. Serial sections of these tissue blocks were alternately stained with Masson’s trichrome or resorcin-fuchsin.

### (3) Morphometry of fibre/structure amounts

For the histomorphometrical analysis, one section stained with Masson’s trichrome and one section stained with resorcin-fuchsin were used of each minipig. The semi-automated morphometrical analysis of the digital microscopic images of these sections was performed with the graphics editing program Adobe Photoshop CS 3 Extended 10.0.1 (Adobe Systems, San Jose, CA, USA). One key feature of this procedure was the placement of pairs of circular Regions of Interest (ROIs) along a midline through each fold (CraF and CauF, see Fig 1); the diameter of each ROI was 35 μm. The midline through the CraF was twice as long as the midline through the corresponding CauF, as the connective tissue of the CraF was thicker (due to the lack of a muscle). Object definition was based on the recognition of the specific colours of collagen structures in Masson’s trichrome stain, and of elastic fibres stained with resorcin-fuchsin. The results of the quantification (measurements) were expressed as amounts per area of collagen structures (apa.coll) and of elastic fibres (apa.elast). The procedure has previously been described in detail [15–16].

### (4) Data analysis

**Fig 2:**
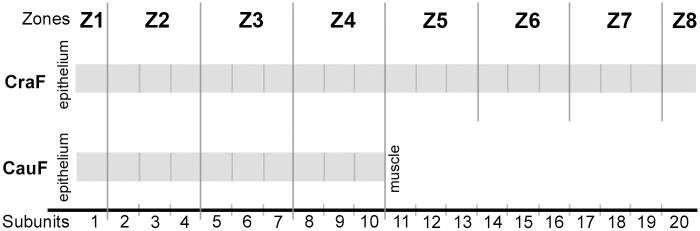
Graphical illustration of the proportional subunits and the zones of the cranial (CraF) and caudal (CauF) fold. Each examined connective tissue section of the CraF was separated into 20 subunits of equal thickness; the connective tissue component of the CauF was separated into 10 subunits due to its smaller thickness. Within the total thickness of the CraF, eight zones (Z1–Z8) were hypothetically defined; accordingly, the total thickness of the CauF was divided into four hypothetical zones (Z1–Z4).

The size, i.e. thickness, of the individual folds was very different. For this reason, the thickness was standardised by subdividing it into proportional subunits of 10% depth each. Starting below the epithelium, 10 subunits were assigned in each CauF (10 subunits—each of 10%—making 100%; see Fig 2). Due to its greater depth, the CraF was subdivided into 20 subunits, as shown in Fig 2. Then, the subunits of the CauF were grouped to form 4 hypothetical zones—zone 1 (Z1) to zone 4 (Z4)—according to Lang et al. [15]. Respecting the greater thickness of the CraF, 4 additional zones were arranged (yielding a total of 8 zones, Z1–Z8, in the CraF; see Fig 2).

The amounts of collagen structures and elastic fibres (apa.coll, apa.elast) were then plotted in diagrams according to the format described above (Fig 2). Statistical analyses of differences in apa.coll and apa.elast were performed between different locations within the same age group, and between the age groups. The analysis of differences within the same age group was performed by pairwise comparison of single subunits with their neighbouring subunits, and by comparing groups of subunits. The analysis of differences between the age groups was performed by pairwise comparison of the equivalent zones of each age group, for example: Apa.coll in zone 1 (Z1) of the ‘young’ group was compared with apa.coll in zone 1 (Z1) of the ‘old’ group. Model residuals were tested for normal distribution using the Kolmogorov-Smirnov test, and by visual assessment of q-q-plots. As the majority of tested data sets were not normally distributed, distribution free non-parametric methods were used: Differences within the same age group (paired samples) were calculated by the Wilcoxon signed-rank test for paired observations, considering the comparison-wise error rate. An α-adjustment for experiment-wise errors was not applied, as the risk of increasing type 2 errors appeared higher than the potential of minimising type 1 errors, particularly as each sample group was compared to a maximum of two other groups. Differences between age groups (independent samples) were calculated using the Kruskal-Wallis test. Resulting p-values of p < = 0.05 were regarded as statistically significant. All analyses were performed with the statistical program package SAS (Version 9.3, SAS Institute, Cary, NC, USA).

### (5) Scoring of the diameters of collagen structures

The diameters of collagen structures—i.e. collagen fibres and collagen fibre bundles—were evaluated in all minipigs included in this study. The analysis was performed according to the semiquantitative scoring system described by Lang et al. [15], which had distinguished three size categories and four scores (see Table 1). Scoring was performed on digital images, with pairs of ROIs aligned along a midline as explained above (see Fig 1). The diameter of each ROI was 70 μm. Scoring was performed twice by the same observer with histological expertise and in random order, i.e. under blinded conditions; the scores were then plotted in diagrams.

**Table 1:** Scoring system describing the number of collagen structures (fibres, and fibre bundles) of different diameters.

	Number of objects	Score
Fibres/Small fibre bundles; diameter: 2–4 μm	0	0
	1–4	1
	5–10	2
	>10	3
Intermediate fibre bundles; diameter: >4–10 μm	0	0
	1–3	1
	4–8	2
	>8	3
Large fibre bundles; diameter: >10 μm	0	0
	1–2	1
	3–4	2
	>4	3

## Results

### (1) General observations from microscopical inspection

Three findings related to age differences were particularly noticeable upon microscopical observation of the tissue sections. Firstly, the cell nuclei of fibroblasts were much more plentiful in the ‘young’ animals compared to the ‘old’ group; this was found in all zones of the connective tissue of both CraF and CauF. Secondly, there was an aberrant tissue reaction to the Masson’s trichrome stain in the ‘old’ animals: The collagen structures in zones 1 to 3 (Z1–Z3) of the CraF of this age group were stained reddish, rather than green. Due to this phenomenon, the Object Definition by selection of green structures and the related measurement of collagen structure amounts yielded false negative values, i.e. close to 0% of apa.coll in Z1–Z3 of the CraF. Thirdly, structural changes in the elastic fibres in the ‘old’ animals were noticed: Elastic fibres appeared as clusters/clumps of 5–10 μm diameter, forming focal aggregations in both CraF and CauF.

### (2) Histomorphometry and scoring: CauF, collagen structures

**Fig 3:**
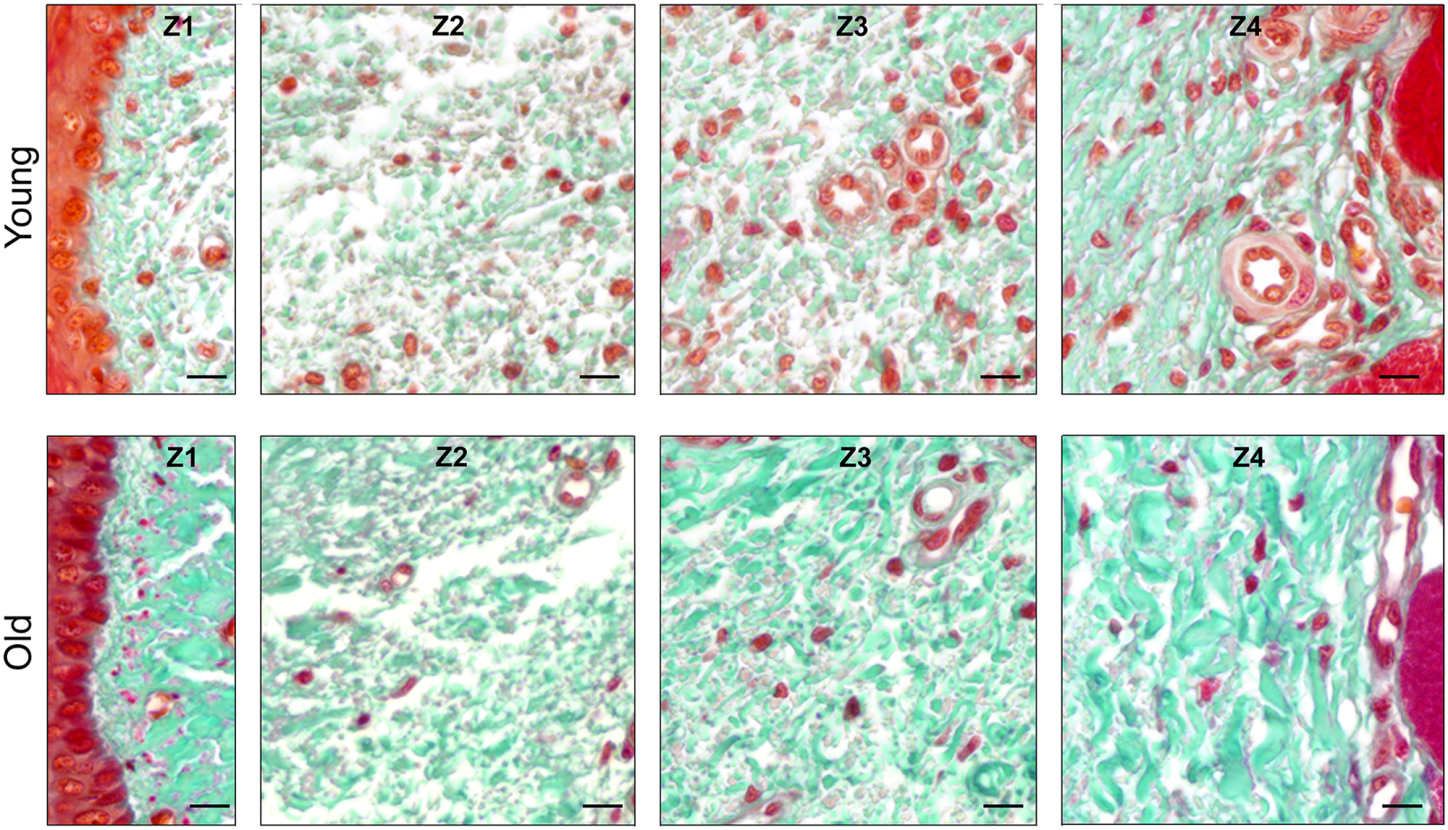
Collagen structures (fibres and fibre bundles) in the CauF of ‘young’ and ‘old’ minipigs (all images in identical magnification). Four zones were defined hypothetically for statistical analysis: Zone 1 (Z1) was a narrow band underneath the epithelium; zones 2 to 4 (Z2–Z4) divided the remaining fold into equidistant thirds. The images serve as examples to illustrate the distinct characteristics of each zone. Note that, according to statistical analysis, not all of them were clearly demarcated. Paraffin section, Masson’s trichrome stain. Bar = 10 μm.

**Fig 4:**
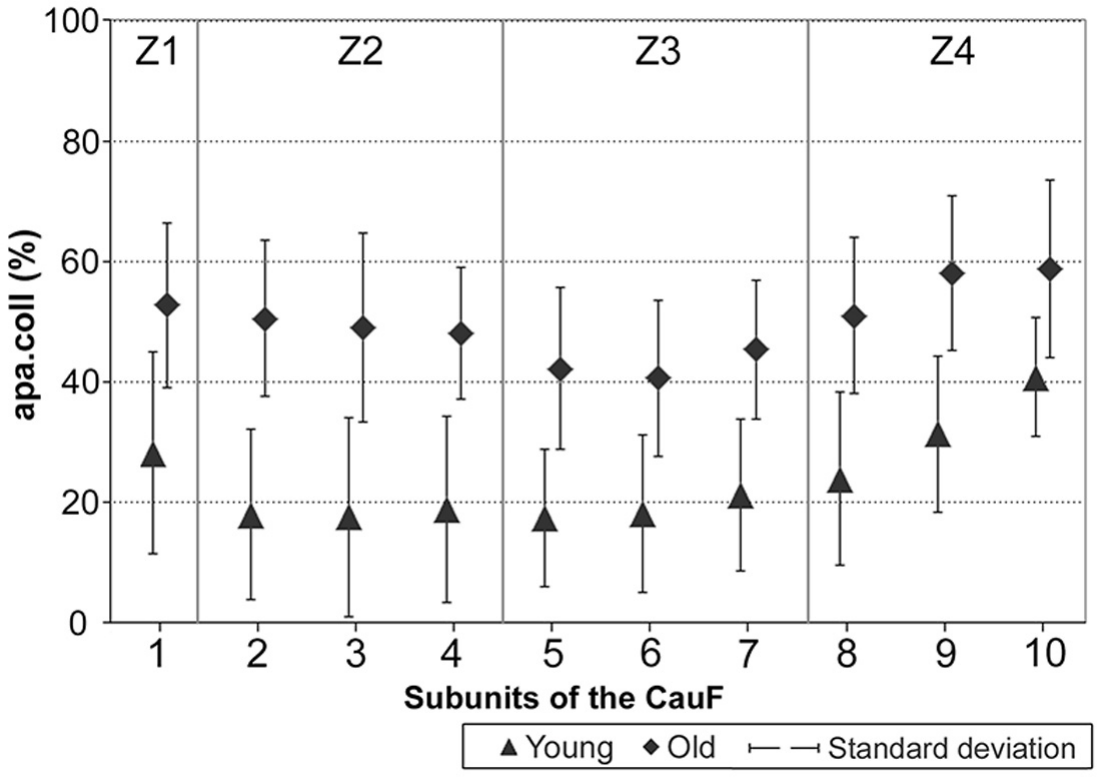
Amounts of collagen structures per area, apa.coll (%), in ‘young’ and ‘old’ minipigs. Four hypothetical zones were assigned in the CauF: Zone 1 (Z1) was a narrow band underneath the epithelium; zones 2 to 4 (Z2–Z4) divided the remaining fold into equidistant thirds.

Within both age groups, the distribution pattern of collagen structures in the CauF was similar (Figs 3 and 4): In zone 1 (Z1), the amounts of collagen structures per area (apa.coll) were always high, but in zones 2 and 3 (Z2, Z3) they were low. In the deep parts of Z3, a continuous re-increase in apa.coll began; finally, subunit 10 of zone 4 (Z4) contained another maximum.

**Fig 5:**
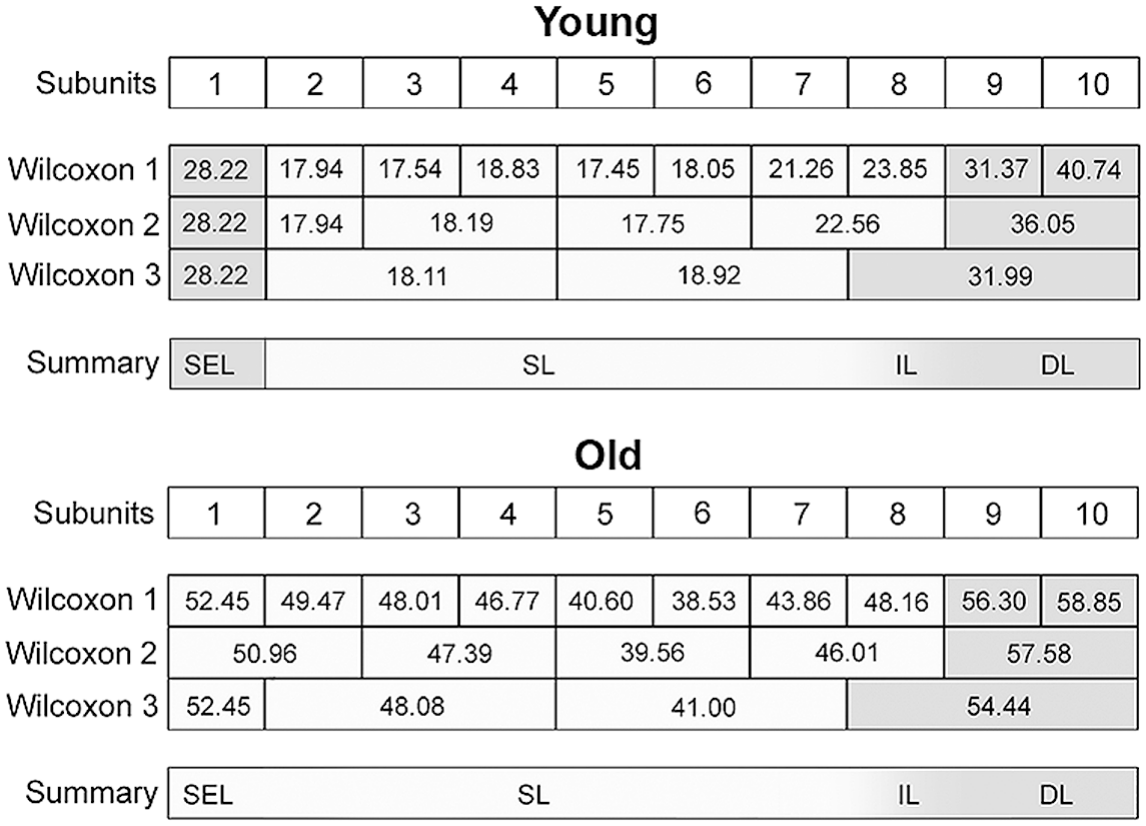
Graphical representation of the statistical analysis (Wilcoxon signed-rank test) of differences in the amounts of collagen structures, i.e. apa.coll (%), in 10 proportional subunits of the CauF. The analyses were performed separately for each age group (‘young’ and ‘old’) and are displayed in three different diagrams. Note: Different designs (i.e. intensities of grey colour) indicate that the differences between the subunits, or groups of subunits, were *statistically significant*. The amounts of collagen structures (mean values) are recorded in the boxes arranged in each bar. In each age group, three procedures of comparison were performed, each displayed in one ‘Wilcoxon Bar’. Wilcoxon Bar 1: Comparison of one subunit with its preceding and succeeding neighbour. Wilcoxon Bar 2: Comparison of groups made up of 2 subunits each. Subunit 1 was not grouped with its neighbouring subunit 2 if its values were significantly higher than those of subunit 2. Wilcoxon Bar 3: Comparison of groups representing the hypothetical zones 1 to 4 (see Figs 2 and 4). Bar 4 summarises bars 1–3; here, a *shaded* design indicates *continuous* transitions, because the distinct demarcation lines (representing statistically significant differences) were located differently in bars 1, 2, and 3, respectively.

**Fig 6:**
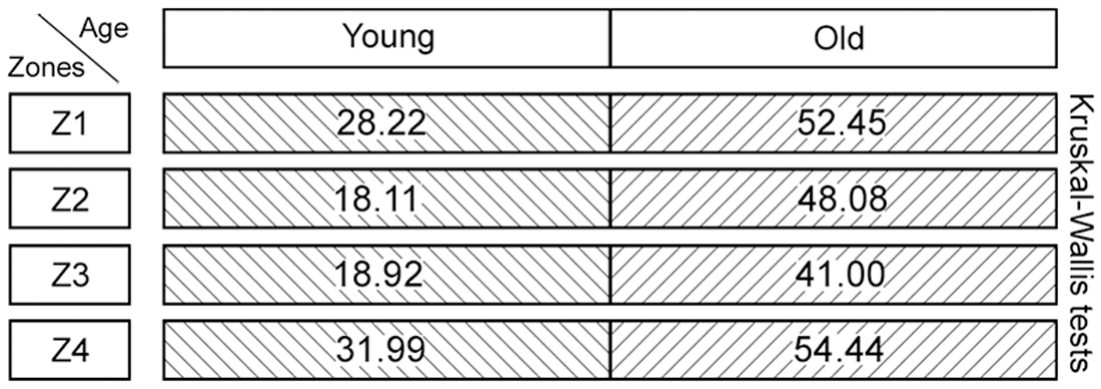
Graphical representation of the statistical analysis (Kruskal-Wallis test) of differences in the amounts of collagen structures in the hypothetical zones 1 to 4 (Z1–Z4) between the two age groups (‘young’ versus ‘old’). These pairwise comparisons are displayed as two adjoining boxes. Note: Different designs (orientation of lines) indicate that the differences between the age groups were *statistically significant*.

With age, there was an increase in apa.coll in the CauF (Figs 3–5), which was statistically significant in all zones of the fold (Fig 6). This increase in apa.coll led to a partial loss of the layered organisation: A demarcation between a subepithelial layer (SEL) and a superficial layer (SL) was no longer statistically detectable in the ‘old’ animals (Fig 5).

**Fig 7:**
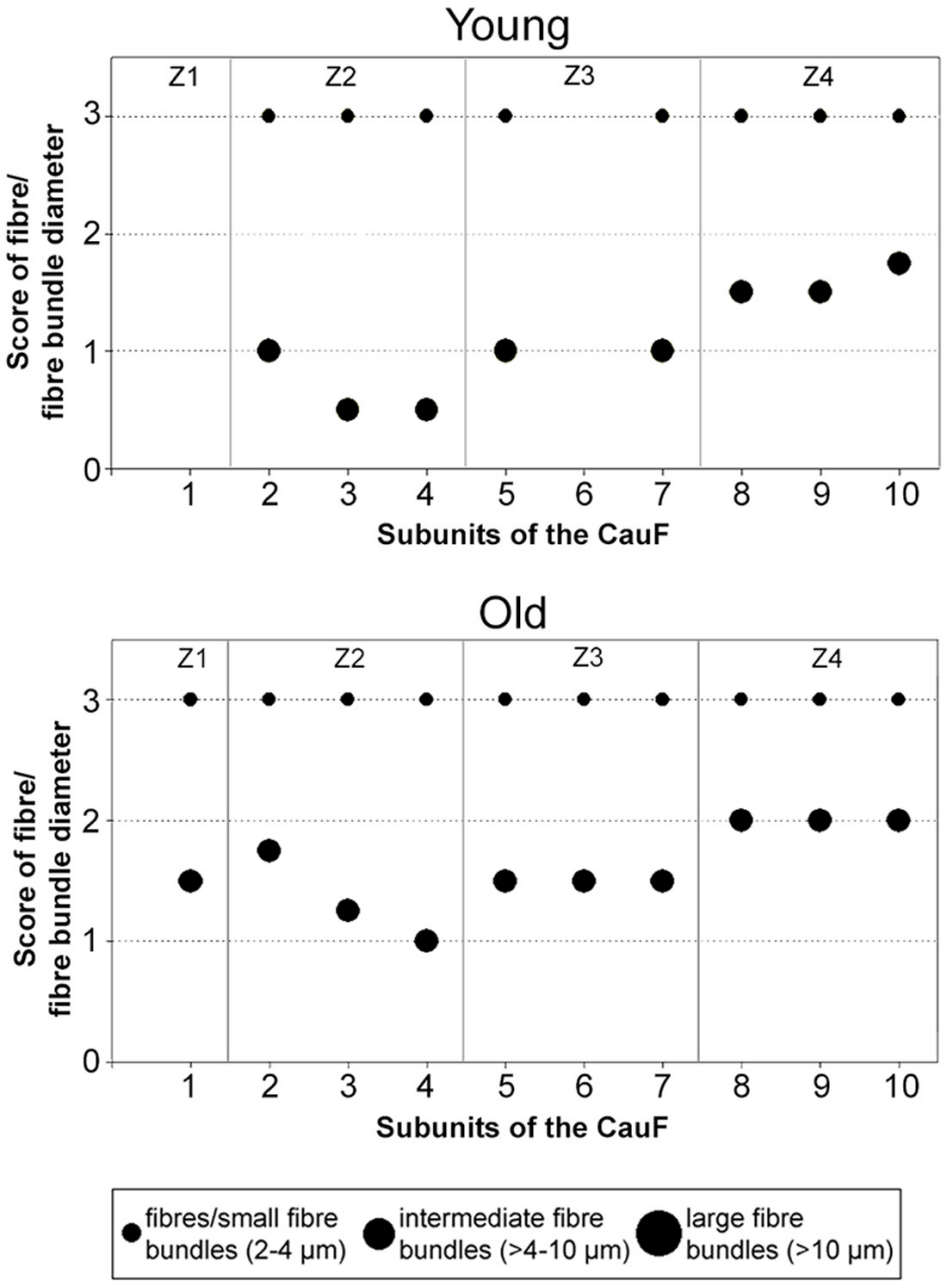
Scores of collagen fibres and fibre bundles of different sizes (diameters) in the CauF of ‘young’ and ‘old’ minipigs. Four hypothetical zones were assigned in the CauF: Zone 1 (Z1) was a narrow band underneath the epithelium; zones 2 to 4 (Z2–Z4) divided the remaining fold into equidistant thirds. Each dot represents the median score of all animals of an age group. Missing values in the subunits 1 and 6 of the ‘young’ group are due to the small size of the CauF in these animals, which only allowed the placement of a small number of ROI-pairs. Consequently, not every subunit contained a ROI-pair.

The scores of the collagen fibres and fibre bundles in the CauF changed very little with age (Fig 7). Age-related differences were found only in the parameter ‘intermediate size fibre bundles’ (ø > 4–10 μm), coinciding with the above-mentioned age-related increases of apa.coll; in other words: The intermediate fibre bundles caused the increase in apa.coll.

### (3) Histomorphometry: CauF, elastic fibres

**Fig 8:**
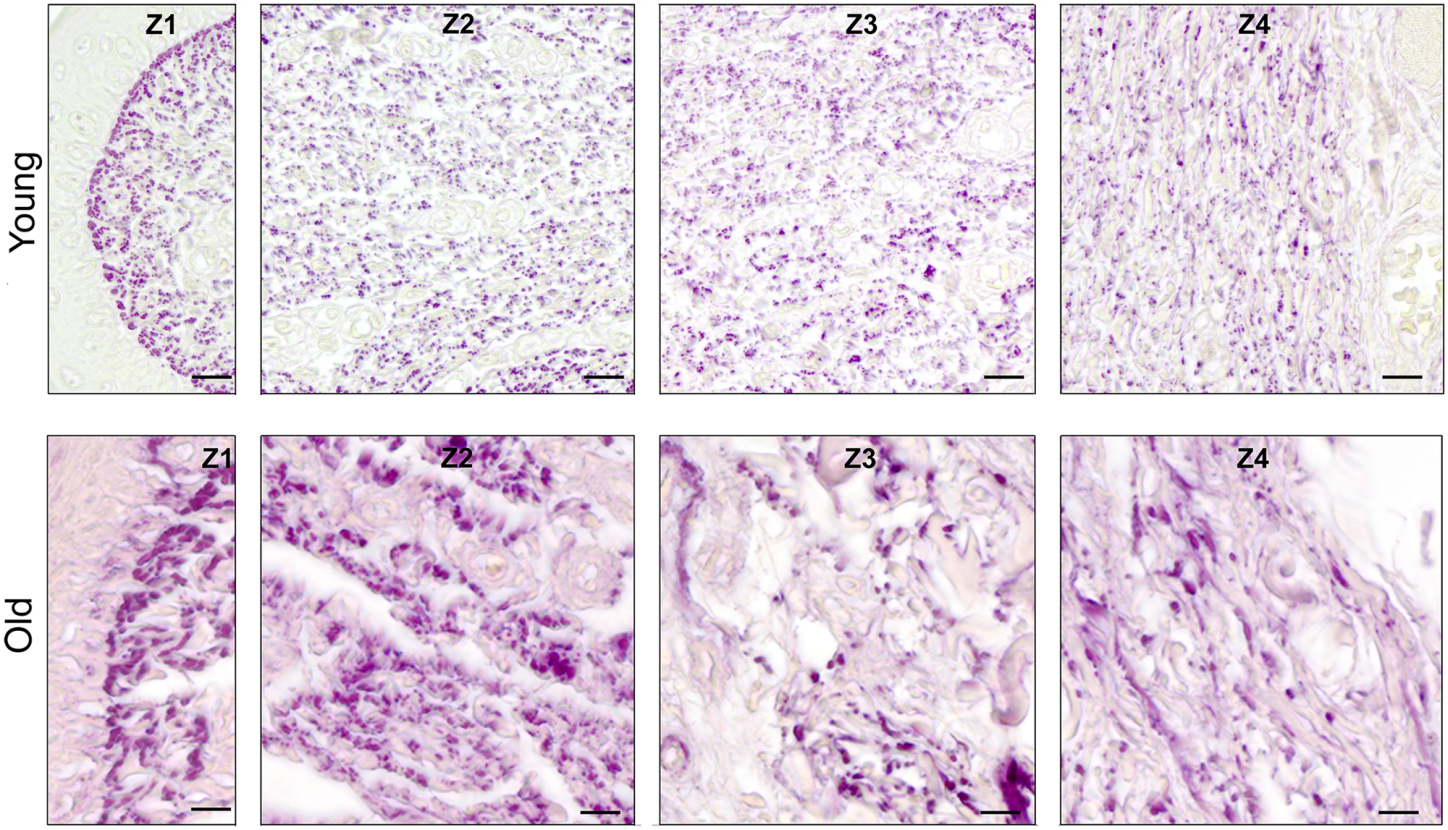
Elastic fibres in the CauF of ‘young’ and ‘old’ minipigs (all images in identical magnification). Four zones were defined as explained in Fig 3. The images serve as examples to illustrate the distinct characteristics of each zone. Note that, according to statistical analysis, not all of them were clearly demarcated. Paraffin section, resorcin-fuchsin stain. Bar = 10 μm.

**Fig 9:**
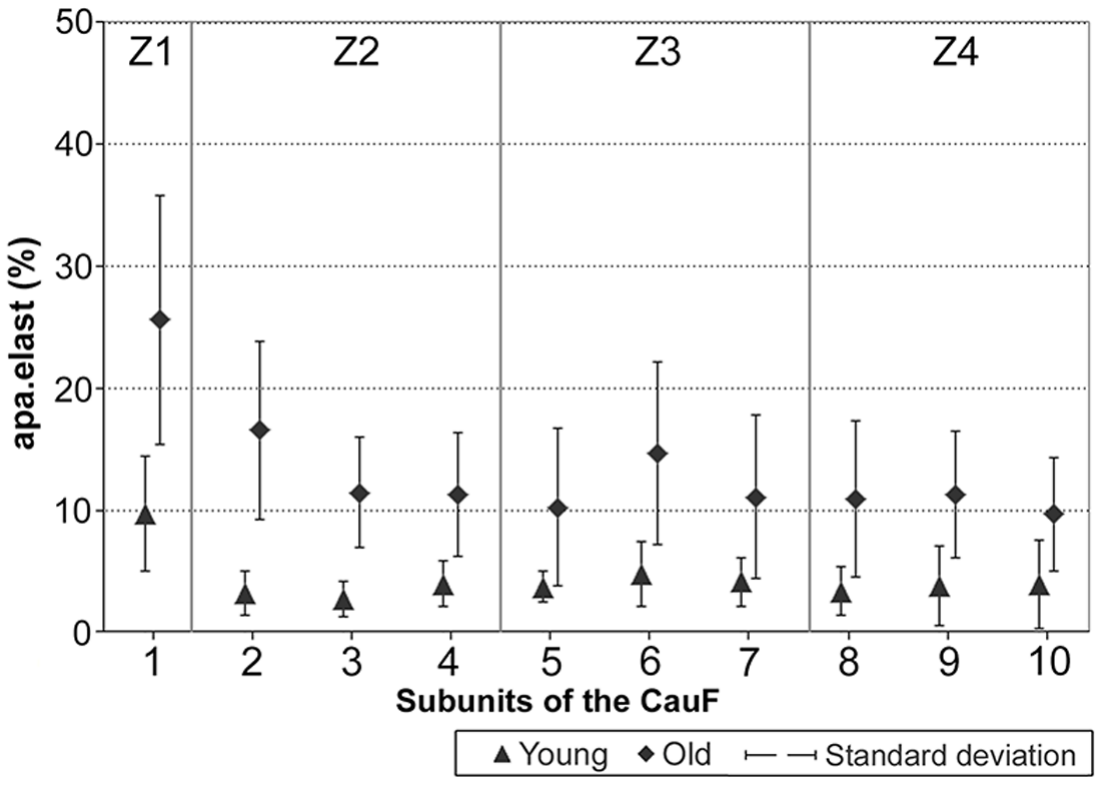
Amounts of elastic fibres per area, apa.elast (%), in ‘young’ and ‘old’ minipigs. Subunits and zones as explained in Fig 4.

The distribution pattern of the elastic fibres was similar within the age groups insofar as in both cases, zone 1 (Z1) was highest in apa.elast, while zones 2 to 4 (Z2–Z4) contained relatively few elastic fibres (Figs 8 and 9).

**Fig 10:**
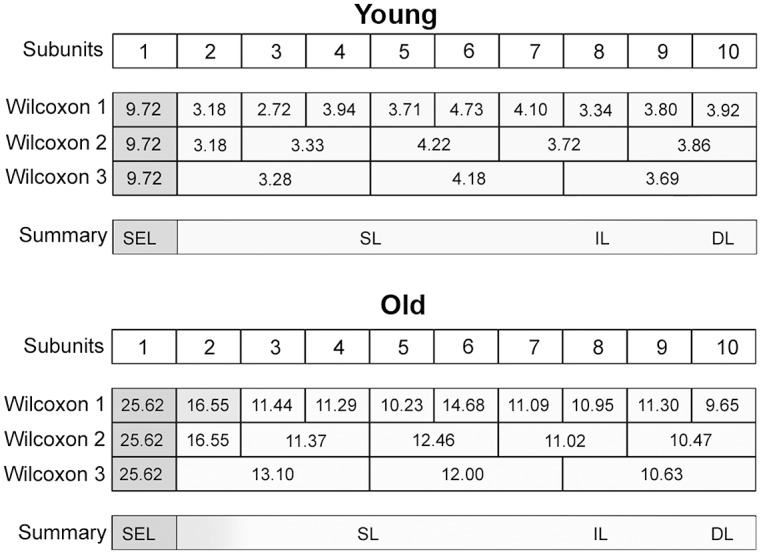
Graphical representation of the statistical analysis (Wilcoxon signed-rank test) of differences in elastic fibre amounts, i.e. apa.elast (%), in 10 proportional subunits of the CauF. The analyses were performed separately for each age group (‘young’ and ‘old’) and are displayed in three different diagrams. Note: Different designs (i.e. intensities of grey colour) of the boxes indicate that the differences between the subunits, or groups of subunits were *statistically significant*. The amounts of elastic fibres (mean values) are recorded in the boxes arranged in each bar. In each age group, three procedures of comparison were performed (‘Wilcoxon Bars’ 1–3), and are summarised in bar 4 (Summary), as explained in Fig 5.

**Fig 11:**
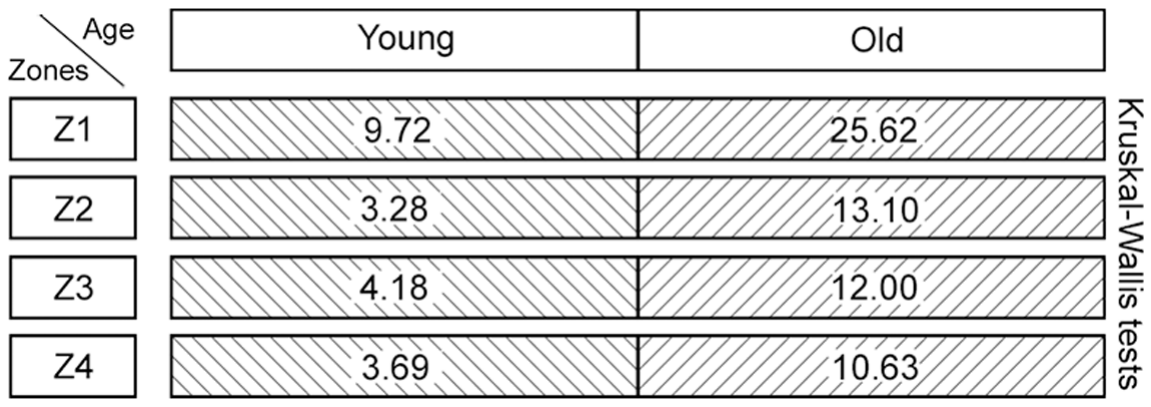
Graphical representation of the statistical analysis (Kruskal-Wallis test) of differences in elastic fibre amounts in the hypothetical zones 1 to 4 (Z1–Z4) between the two age groups (‘young’ versus ‘old’). These pairwise comparisons are displayed as two adjoining boxes. Note: Different designs (orientation of lines) indicate that the differences between the age groups were *statistically significant*.

There was a significant age-related increase in apa.elast throughout all zones of the CauF (Figs 8–11). The strongest increase in apa.elast was found in zone 1 (Z1): 9.72% in the ‘young’ group (mean value; n = 6) versus 25.62% in the ‘old ‘group (mean value; n = 6), i.e. a value more than twice as high (Figs 9 and 10). Age-related differences in apa.elast became slightly less distinct in the depth of the fold.

### (4) Histomorphometry and scoring: CraF, collagen structures

**Fig 12:**
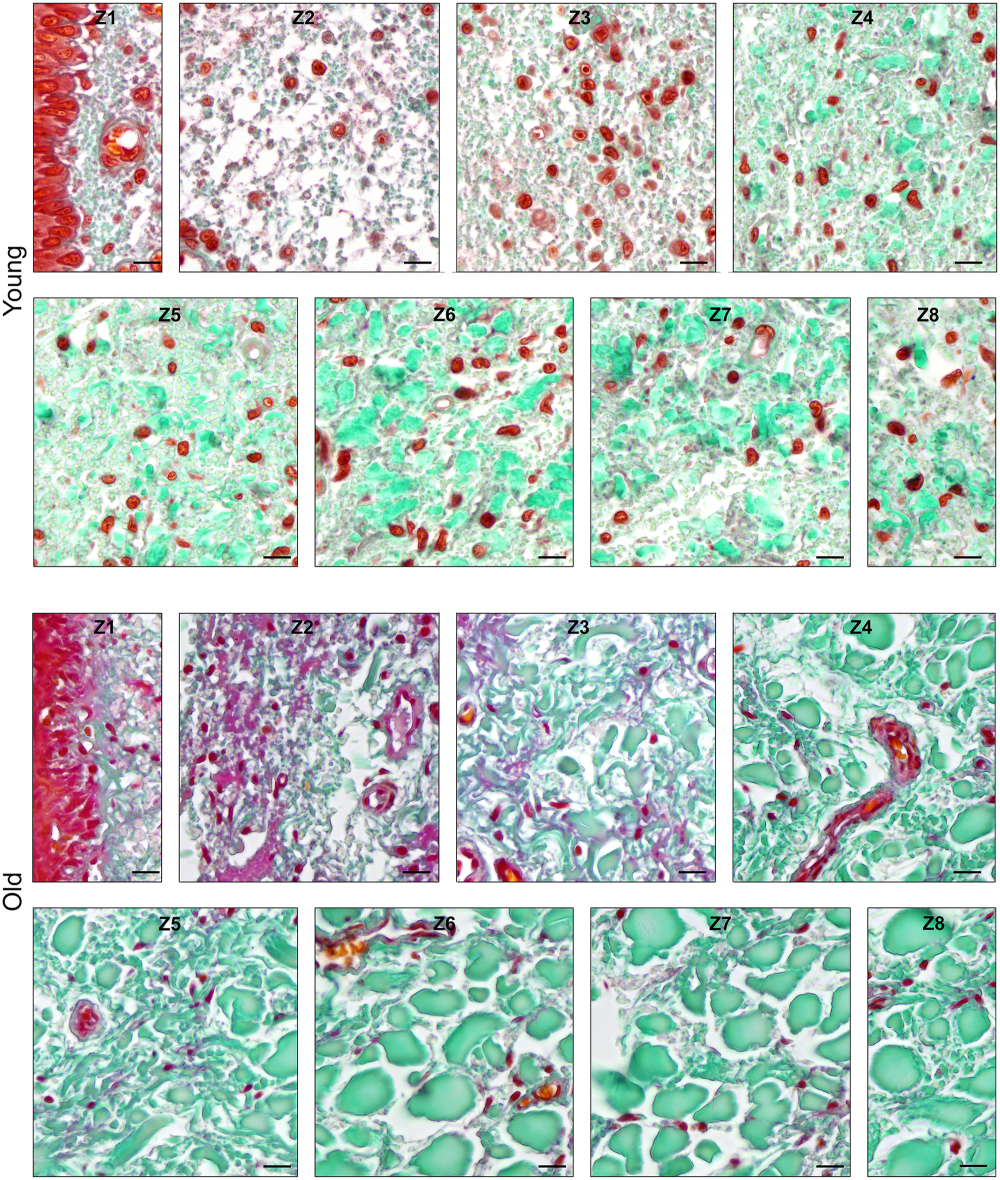
Collagen structures (fibres and fibre bundles) in the CraF of ‘young’ and ‘old’ minipigs (all images in identical magnification). Hypothetical zones were defined for statistical analysis: Zone 1 (Z1) was a narrow band underneath the epithelium (representing subunit 1), zones 2 to 7 (Z2–Z7) each comprised three subunits; zone 8 (Z8) represented the remaining subunit 20. The images serve as examples to illustrate the distinct characteristics of each zone. Note that, according to statistical analysis, not all of them were clearly demarcated. Paraffin section, Masson’s trichrome stain. Bar = 10 μm.

**Fig 13:**
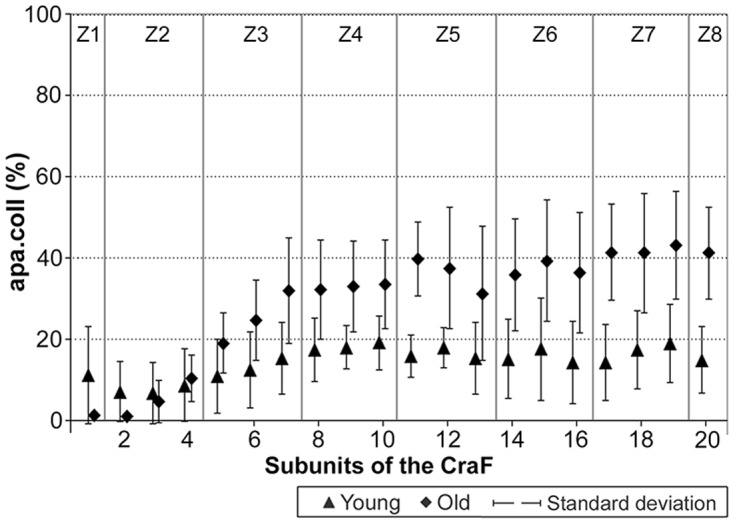
Amounts of collagen structures per area, apa.coll (%), in ‘young’ and ‘old’ minipigs. Eight hypothetical zones were assigned in the CraF: Zone 1 (Z1) was a narrow band underneath the epithelium (representing subunit 1), zones 2 to 7 (Z2–Z7) each comprised three subunits; zone 8 (Z8) represented the remaining subunit 20.

**Fig 14:**
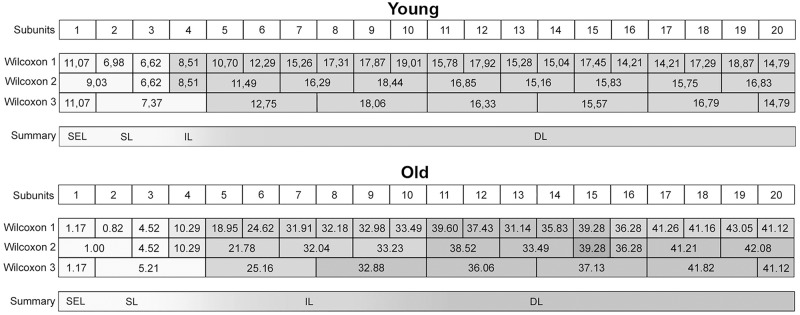
Graphical representation of the statistical analysis (Wilcoxon signed-rank test) of differences in the amounts of collagen structures, i.e. apa.coll (%), in 20 proportional subunits of the CraF. The analyses were performed separately for each age group (‘young’ and ‘old’) and are displayed in three different diagrams. Note: Different designs (i.e. intensities of grey colour) of the boxes indicate that the differences between the subunits, or groups of subunits, were *statistically significant*. The amounts of collagen structures (mean values) are recorded in the boxes arranged in each bar. Three procedures of comparison were performed (‘Wilcoxon Bars’ 1–3), and are summarised in bar 4 (Summary), as explained in Fig 5.

Irrespective of the age-group, the superficial zones of the CraF (Z1–Z3) contained less apa.coll than the deeper zones (Figs 12 and 13): In Z1 of the ‘young’ group, there were slightly more collagen structures than in the neighbouring Z2, indicating the presence of a subepithelial layer (SEL) in this age group. However, this finding was not confirmed by statistical analysis (Fig 14). The ‘old’ group displayed very different values of apa.coll in Z1 and Z2 compared to the remaining CraF: Hardly any collagen was recorded due to a reddish instead of a green stain of the collagen structures (Fig 12).

**Fig 15:**
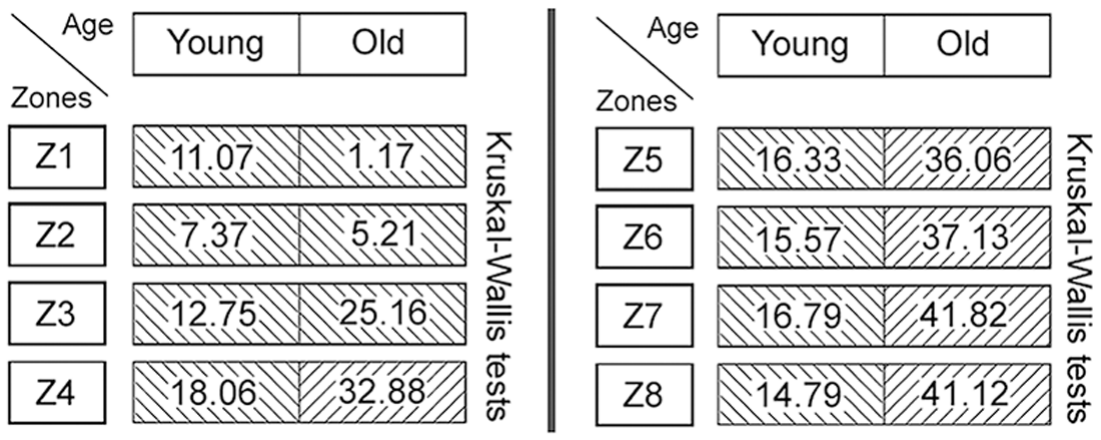
Graphical representation of the statistical analysis (Kruskal-Wallis test) of differences in the amounts of collagen structures in the hypothetical zones 1 to 8 (Z1–Z8) between the two age groups (‘young’ versus ‘old’). These pairwise comparisons are displayed as two adjoining boxes. Note: Different designs (orientation of lines) indicate that the differences between the age groups were *statistically significant*.

An age-related increase in apa.coll was shown by comparing the ‘young’ and the ‘old’ group (Figs 13 and 15). Apa.coll was approx. doubled in all regularly stained zones (Z3–Z8) of the CraF (Fig 13), which was statistically significant in zones 4 to 8 (Z4–Z8) (Fig 15).

**Fig 16:**
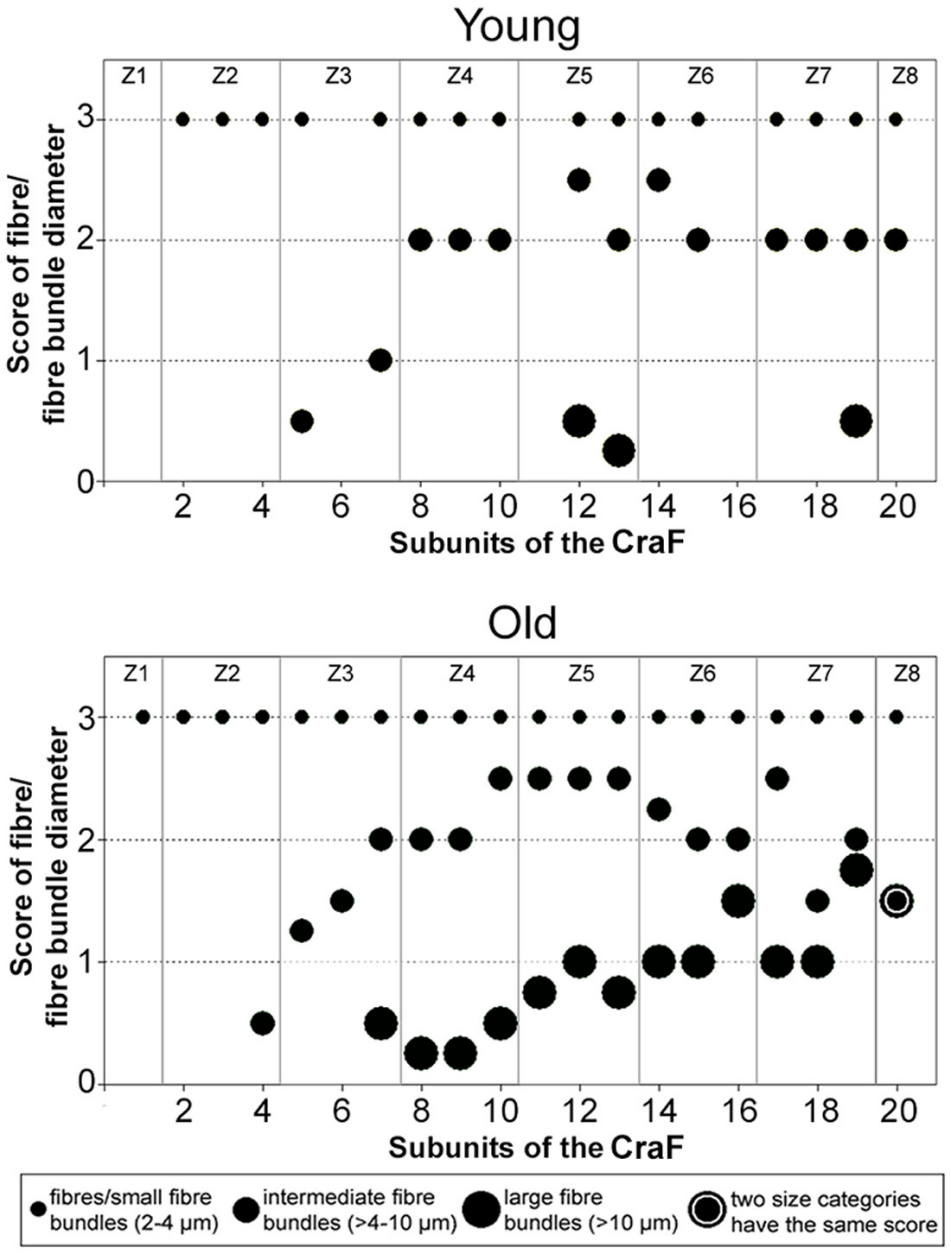
Scores of collagen fibres and fibre bundles of different sizes (diameters) in the CraF of ‘young’ and ‘old’ minipigs. Eight hypothetical zones were assigned in the CraF: Zone 1 (Z1) was a narrow band underneath the epithelium (representing subunit 1), zones 2 to 7 (Z2–Z7) each comprised three subunits; zone 8 (Z8) represented the remaining subunit 20. Each dot represents the median score of all animals of an age group. Missing values in the subunits 1, 6, 11 and 16 of the ‘young’ group are due to the small size of the CraF in these animals, which only allowed the placement of a small number of ROI-pairs. Consequently, not every subunit contained a ROI-pair.

Both the number and the diameters of the bundles of collagen structures increased with age (Fig 16): In the ‘young’ group, large collagen fibre bundles (>10 μm) were very rare, but their number increased in the ‘old’ animals, particularly in the intermediate and deep zones (Z1–Z8). The scores of the intermediate fibre bundles were also slightly higher in the ‘old’ animals compared to the ‘young’ ones (Fig 16).

### (5) Histomorphometry: CraF, elastic fibres

**Fig 17:**
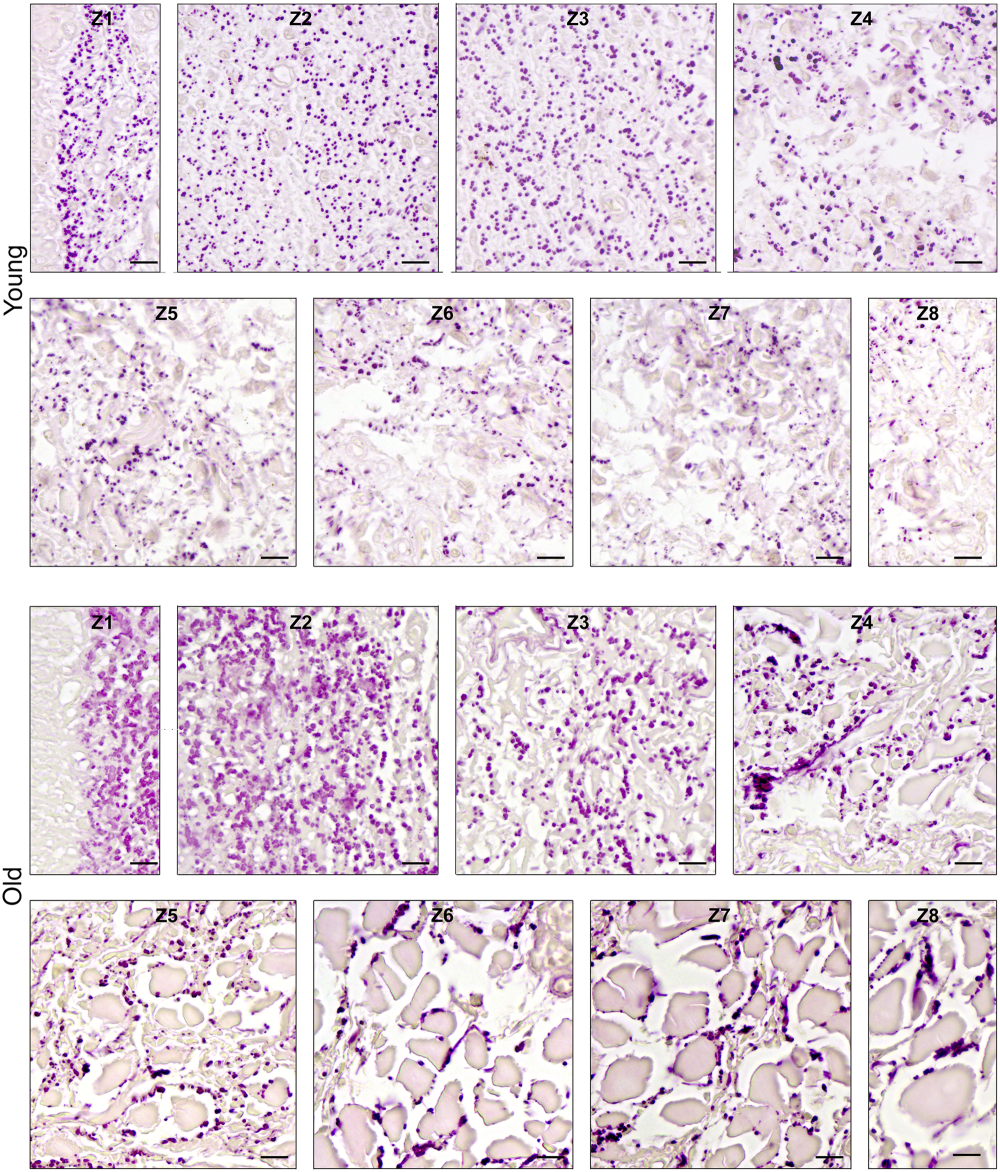
Elastic fibres in the CraF of ‘young’ and ‘old’ minipigs (all images in identical magnification). Eight zones were defined as explained in Fig 12. The images serve as examples to illustrate the distinct characteristics of each zone. Note that, according to statistical analysis, not all of them were clearly demarcated. Paraffin section, resorcin-fuchsin stain. Bar = 10 μm.

**Fig 18:**
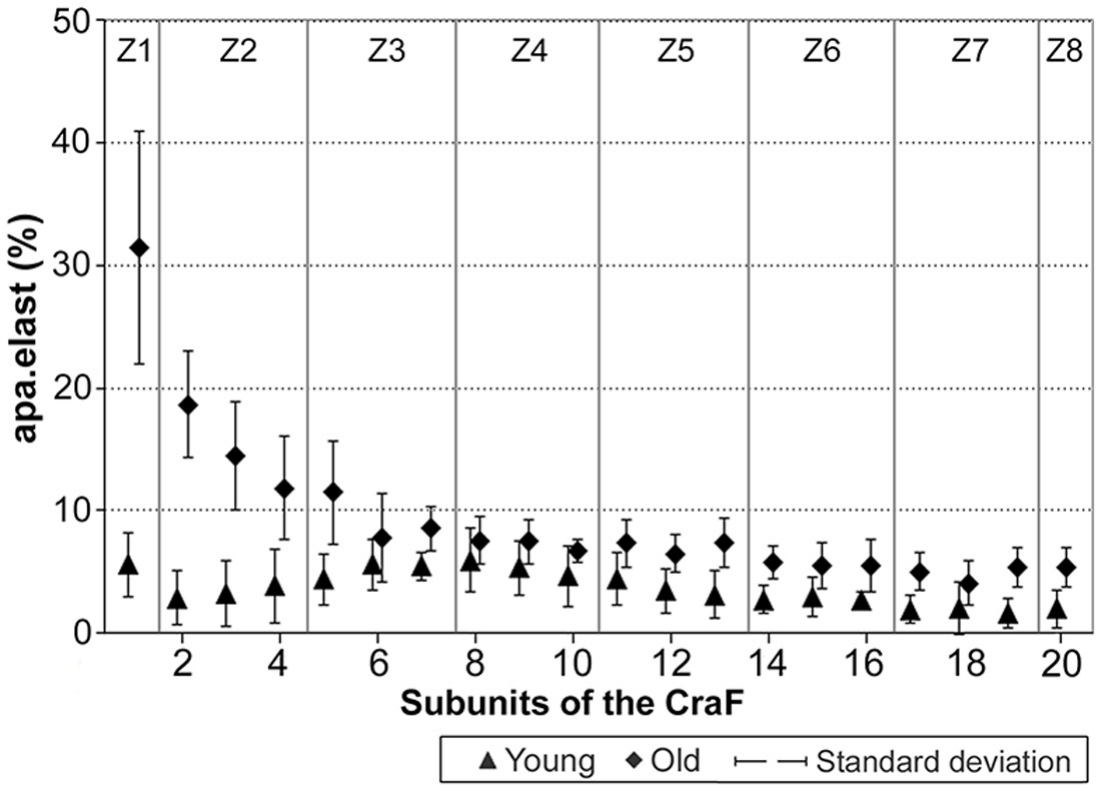
Amounts of elastic fibres per area, apa.elast (%), in ‘young’ and ‘old’ minipigs. Eight hypothetical zones were assigned in the CraF: Zone 1 (Z1) was a narrow band underneath the epithelium (representing subunit 1), zones 2 to 7 (Z2–Z7) each comprised three subunits; zone 8 (Z8) represented the remaining subunit 20.

**Fig 19:**
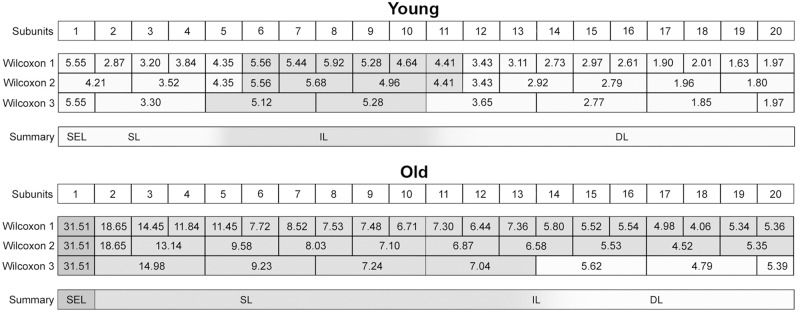
Graphical representation of the statistical analysis (Wilcoxon signed-rank test) of differences in elastic fibre amounts, i.e. apa.elast (%), in 20 proportional subunits of the CraF. The analyses were performed separately for each age group (‘young’ and ‘old’) and are displayed in three different diagrams. Note: Different designs (i.e. intensities of grey colour) indicate that the differences between the subunits, or groups of subunits, were *statistically significant*. The elastic fibre amounts (mean values) are recorded in the boxes arranged in each bar. Three procedures of comparison were performed (‘Wilcoxon Bars’ 1–3), and are summarised in bar 4 (Summary), as explained in Fig 5.

**Fig 20:**
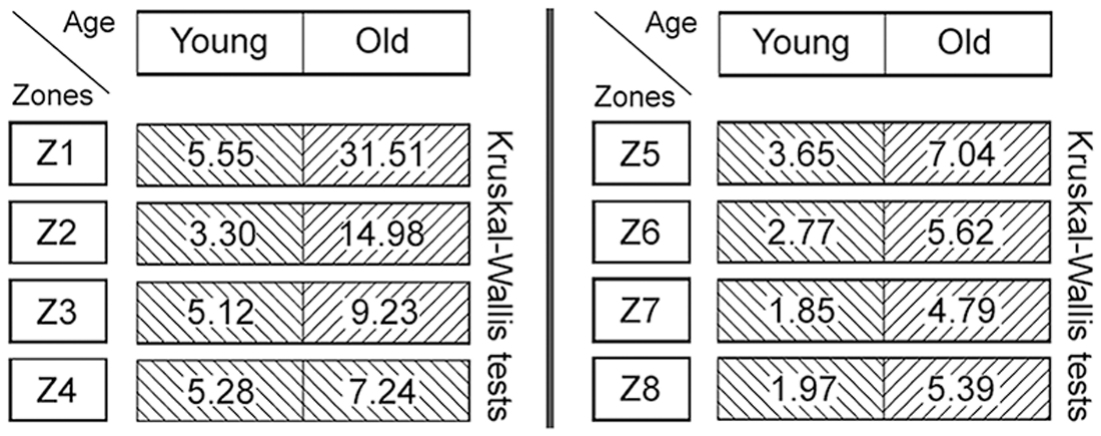
Graphical representation of the statistical analysis (Kruskal-Wallis test) of differences in elastic fibre amounts in the hypothetical zones 1 to 8 (Z1–Z8) between the two age groups (‘young’ versus ‘old’). These pairwise comparisons are displayed as two adjoining boxes. Note: Different designs (orientation of lines) indicate that the differences between the age groups were statistically significant.

Two features concerning the elastic fibres in the CraF were particularly distinct: Firstly, there was a strong age-related increase in apa.elast in zones 1 and 2 (Z1, Z2) (Figs 17–20). In Zone 1, apa.elast increased from 5.55% (mean value; n = 6) in the ‘young’ animals to approx. six times this amount (mean value: 31.51%; n = 6) in the ‘old’ minipigs (Fig 18). Secondly, there was a distinct layer of relatively high apa.elast located in zones 3 and 4 (Z3–Z4) in the ‘young’ group. Consequently, a clear distinction could be made between a superficial layer (SL, low in elastic fibres), an intermediate layer (IL, containing significantly higher amounts of elastic fibres), and a deep layer (DL; again low in apa.elast) in this group of ‘young’ minipigs (Figs 17 and 19).

The entire scope of our findings was very complex and heterogeneous; the most striking findings are, therefore, compiled in Table 2.

**Table 2:** Survey on the most prominent findings concerning collagen structures and elastic fibres in the cranial (CraF) and caudal (CauF) vocal fold of minipigs of different ages.

**General observations**	
Age-related increase in amounts of apa.coll and apa.elast in both CraF and CauF (see details below)	
Stratigraphical arrangement of collagen structures not identical with that of elastic fibres	
**CauF**	
*Collagen structures*	*Elastic fibres*
In the ‘old’ minipigs loss of a clear, layered structure	Strong increase in the superficial zones of the ‘old’ group
Diameters remained small	
**CraF**	
*Collagen structures*	*Elastic fibres*
Similar layered structure present in both age groups	Strong increase in the superficial zones of the ‘old’ group
Increase in apa.coll in the intermediate and deep layers (IL, DL)	In the ‘young’ group: Intermediate layer (IL) clearly distinct from superficial and deep layers
Increase of both diameter and number of collagen structures	
**Comparison CauF versus CraF**	
*Texture and density of tissue*	
Always more fibres (collagen, elastic) in the CauF than in the CraF	
*Collagen structures*	
Growth of fibre bundles topographically restricted to the deep zones of the CraF	
*Elastic fibres*	
Elastic components in the intermediate layer: Distinct characteristic of the CraF of the ‘young’ minipigs	

## Discussion

### Structural considerations—Definition and distinction of layers

The findings of the visual (microscopical) inspections and of the morphometrical studies, acquired with reference to the different topographical zones, will be discussed in order to confirm or reject the presence of a characteristic stratigraphical organisation of the lamina propria of the vocal folds of minipigs. Three criteria will be considered: (1) Amounts of fibres (Quantity); (2) Types of fibres (Quality); (3) Demarcation (Distinct versus continuous transitions).

One of the most striking *common features* of CraF and CauF was the presence of the relatively *dense*, *collagenous-elastic subepithelial layer* (SEL). The SEL had already been described in 6–12 month old pigs (breed not mentioned) by Hahn et al. [27–28], and in ‘adult’ minipigs (11–27 months) by Lang et al. [15], and has now also been detected in minipigs of other ages: It was obvious already in the youngest (2–3 months) of our minipigs, and, in principle, also in the ‘old’ animals (4–7 years). An exception to this rule was found only in the CraF of the ‘old’ minipigs: Here, the contribution of collagen fibres to this SEL seemed to be low. However, this surprising finding may be related to a technological phenomenon: In Masson’s trichrome, the fibres in this location were stained reddish (instead of green). Staining results remained the same despite several modifications of the Masson’s trichrome staining protocol (as described by Lang et al. [15]). Obviously, their microstructural properties, i.e. fibre quality—in this location, at this age—differed from those found in the other age groups, and the variation of data was related to differences in quality rather than to differences in quantity of collagen fibres in the SEL. Therefore, an attempt to adjust our procedure of Object Definition to recognise the reddish fibres did not appear appropriate; the reddish fibres were thus discriminated in our procedure of Object Definition. Further tests, i.e. immunohistochemical examinations, are in progress to elucidate this problem. Until then, it remains unclear why this atypical phenomenon occurred only in the CraF.

Lang et al. [15] stressed, in a topographical sense, some similarities between the subepithelial layer (SEL) in the minipig and the so-called basement membrane zone (BMZ) of the human vocal fold, as described by Hammond et al. [22], Hahn et al. [27–28], and Tateya et al. [33]. However, the minipig’s SEL appears much more elastic in structure compared to the primarily collagenous BMZ [33] of humans. The functional impact of this finding will be discussed below (see Functional considerations—The phoniatrical body-cover model).

With increasing age, the loose superficial layer (SL) found in both folds of the ‘young’ animals changed with respect to *fibre quality*, but this change differed in CraF and CauF: This development yielded a predominantly elastic composition of the SL of the CraF, and a mixed (collagenous-elastic) composition of the SL in the CauF. However, in *both* folds the loose character of the SL and therefore also its demarcation from the SEL did no longer exist in the ‘old’ minipigs. The findings of Lang et al. [15] in ‘adult’ minipigs (11–27 months) revealed that the SL of both CraF and CauF was still loose during adulthood. Accordingly, the loss of the loose character of the SL appears to be a result of the process of *ageing* (rather than of maturation, i.e. changes from young to adult states [15]).

In this sense, the SL of the ‘adult’ [15] and ‘old’ minipigs differed distinctly from that of adult and old humans: In humans, the maturation process does not cause any very drastic increase in fibre amounts in the respective proportional depth of the lamina propria; only in the old (‘geriatric’) vocal folds does the ageing process cause a marked increase in fibre density [7, 22–23]. No reports are available concerning a loss of demarcation between BMZ and superficial layer in humans; on the contrary, there are reports of even an increase in width of this loose tissue formation (beginning at approx. 50 years of age [19]). Again, the functional significance of these obvious structural differences between minipigs and humans will be discussed below.

With regard to the stratigraphical organisation of the lamina propria, the *intermediate layer (IL)* deserves special attention: Only in the *CraF of the* ‘*young’ minipigs* was this layer distinctly *demarcated* due to relatively high amounts of *elastic* fibres—a situation similar to that in humans [7, 27, 34]. In contrast, the CraF of the ‘old’ minipigs included in this study, and also of the ‘adult’ minipigs investigated by Lang et al. [15], was characterised by a topographical gradient of elastic fibre content: High in subepithelial locations, and continuously lower towards deeper locations of the folds.

A further *difference* between CraF and CauF—with some relevance for a comparison with humans—became obvious when looking at the lamina propria as a whole, i.e. along its entire depth, and again including the findings of the ‘adult’ minipigs [15] into the consideration: In the CraF, collagen fibre amounts and distribution in the ‘adult’ minipigs [15] were very similar to those of the ‘old’ animals of the present study. Accordingly, the increase in collagen fibre amounts had been completed by the time of adulthood; it was part of the processes of *maturation*. The stratigraphical arrangement in the CraF of the ‘adult’ animals was then preserved in the ‘old’ stage throughout the entire lamina propria. In the CauF, however, the increase of density from ‘young’ to ‘adult’ minipigs [15] continued even in the ‘old’ group, as shown in the present study. This development resulted in the loss of a stratigraphical arrangement, and can be assigned to the processes of *ageing*. Such features of the CauF have been described as a status of the ‘geriatric’ vocal fold in humans [23].

Considering the existence of layers of the lamina propria, our findings were very much in accordance with Infusino et al. [35], who stressed the difficulty of distinguishing a layered structure on the basis of only a subjective inspection of histological sections.

### Functional considerations—The phoniatrical body-cover model

Hirano [34] compared the biomechanical properties of the ‘cover’ to those of soft gelatine. However, one should keep in mind that in the minipigs such a loose tissue layer was separated from the surface epithelium by a *fibro-elastic subepithelial layer* (SEL), which was placed over the loose, i.e. soft parts like a *membrane*. A similar structure in the same location also exists in humans; here, it is called basement membrane zone (BMZ) [22, 27–28, 33]. Yet, there is no data on its dimensions (in humans), and it has not yet been incorporated into the body-cover model. Hirano [34] focused only on the function of the epithelium as a thin and stiff ‘capsule’ whose purpose was to maintain the shape of the vocal fold. Such a function may not be accomplished by the thin layer of epithelial cells alone (in our minipigs, the epithelium of CraF and CauF was only sparsely keratinised; unpublished data), but may require a ‘fortification’ by something like the human BMZ or the porcine SEL. Considering the substantial thickness of the porcine SEL of approx. 20 μm, this membrane-like structure can be assumed to have an impact on the shape of the vocal folds and thus influence its oscillatory characteristics.

The ageing processes of a connective tissue system are characterised, amongst other features, by an increase of the number of cross-links between fibres (both collagen and elastic), which accounts for an increase in tissue stiffness [36] and—with regard to the elastic fibres [37]—a loss of tissue elasticity. Consequently, one may suppose that the formerly soft (and pliable) ‘cover’ in the ‘young’ minipigs (2–3 months) has become stiff and less elastic in the ‘old’ minipigs (aged 4–7 years). These changes appear much more pronounced in the minipigs compared to humans, as in the latter, the cover’s structure remains relatively loose (as mentioned previously).

To date, there is no unanimous agreement about the functional role of the *intermediate layer* of the lamina propria, because it has been assigned by different authors (e.g. [1–2, 4, 6–7]) either to ‘cover’, or ‘transition’, or ‘body’. Our study does not contribute any clarification to this matter. However, it emphasises that such a distinct formation was present only in the CraF of the *‘young’* minipigs. High elasticity and low stiffness [2]—characteristics like in soft rubber bands [34]—are functional key features that might also be attributed to this formation in the CraF of the minipigs. Accordingly, this distinct structural item may be related to the CraF’s role, which has been proposed by Alipour and Jaiswal [12–13] as the main oscillator of the porcine glottis.

In the CraF, the phoniatrical ‘*body’* is completely fibrous, instead of being partly muscular as in the CauF; the thick collagen fibre bundles making up this fibrous ‘body’ have previously been described in ‘adult’ minipigs aged 11–27 months [15]. When correlating these findings with the lack of thick fibre bundles in the ‘young’ minipigs (aged 2–3 months) of the present study, one can assume that the development of these thick collagen fibre bundles—substituting the muscle—is part of the *maturation* processes, rather than part of the processes of ageing. This chronobiological feature emphasises the great functional importance of the thick fibre bundles in the CraF, and of the special phoniatrical properties of the CraF as a whole.
